# Transcriptomic Analysis of Early Flowering Signals in ‘Royal’ Flax

**DOI:** 10.3390/plants11070860

**Published:** 2022-03-24

**Authors:** Megan A. House, Lester W. Young, Stephen J. Robinson, Helen M. Booker

**Affiliations:** 1 Department of Plant Sciences, University of Saskatchewan, 51 Campus Drive, Saskatoon, SK S7N 5A8, Canada; megan.house@usask.ca (M.A.H.); lester.young@usask.ca (L.W.Y.); 2 Agriculture and Agri-Food Canada, Saskatoon Research and Development Centre, 107 Science Place, Saskatoon, SK S7N 0X2, Canada; steve.robinson2@agr.gc.ca; 3 Department of Plant Agriculture, Ontario Agricultural College, University of Guelph, 50 Stone Rd E, Guelph, ON N1G 2W1, Canada

**Keywords:** flax, RNA-seq, flowering, transcriptome, breeding

## Abstract

Canada is one of the world’s leading producers and exporters of flax seed, with most production occurring in the Prairie Provinces. However, reduced season length and risk of frost restricts production in the northern grain belt of the Canadian Prairies. To expand the growing region of flax and increase production in Canada, flax breeders need to develop earlier-flowering varieties capable of avoiding the risk of abiotic stress. A thorough understanding of flowering control of flax is essential for the efficient breeding of such lines. We identified 722 putative flax flowering genes that span all major flowering-time pathways. Frequently, we found multiple flax homologues for a single Arabidopsis flowering gene. We used RNA sequencing to quantify the expression of genes in the shoot apical meristem (SAM) at 10, 15, 19, and 29 days after planting (dap) using the ‘Royal’ cultivar. We observed the expression of 80% of putative flax flowering genes and the differential expression of only 30%; these included homologues of major flowering regulators, such as *SOC1*, *FUL,* and *AP1*. We also found enrichment of differentially expressed genes (DEGs) in transcription factor (TF) families involved in flowering. Finally, we identified the candidates’ novel flowering genes amongst the uncharacterized flax genes. Our transcriptomic dataset provides a useful resource for investigating the regulatory control of the transition to flowering in flax and for the breeding of northern-adapted varieties.

## 1. Introduction

Canada is one of the world’s leading producers and exporters of flax (*Linum usitatissimum* L.), with most production occurring in the Prairie Provinces [1]. However, environmental constraints limit the growing area of flax to the southern regions of Alberta, Saskatchewan, and Manitoba. Currently, the northern Prairies are not suitable for growing flax because of the short growing season and subsequent risk of an early frost occurring before crop maturation. To expand the growing range of flax, it is necessary to develop cultivars that reach crop maturity within the seasonal constraints of northern prairie environments. Breeding for northern-adapted traits, such as earlier flowering and maturity, and/or day-length neutrality for floral induction, will aid in expanding the northern growing range of flax in Canada.

To improve breeding efforts aimed at developing northern-adapted flax, we first need to understand its genetic flowering mechanisms. The genetic networks underlying these mechanisms are incredibly complex [2,3]. In Arabidopsis, more than 300 genes work together in eight main pathways to coordinate the timing of the reproductive transition [4,5]. A breadth of flowering gene data exists for many species; however, the dissection of specific flowering-time pathways in flax is in its infancy. Flax is a facultative long-day plant, meaning that flowering will occur in non-inductive short days; however, its initiation is earlier with a long photoperiod [6,7]. We previously identified flax homologues of several key photoperiod pathway genes using gene-specific PCR primers [7]. These include homologues of a *CONSTANS-like* (*COL*) gene, *FLOWERING LOCUS T* (*FT*), and *GIGANTEA* (*GI*). Other studies also describe a small number of flax flowering gene homologues from additional induction pathways. These include homologues of *LEAFY* (*LFY*) and *TERMINAL FLOWER 1* (*TFL1*) [8], *SUPPRESSOR OF OVEREXPRESSION OF CONSTANS 1* (*SOC1*), *ADP GLUCOSE PYROPHOSPHORYLASE1* (*ADG1*), *GIBBERELLIC ACID INSENSITIVE* (*GAI*), *APETALA1* (*AP1*) [6], and *TIMING OF CAB EXPRESSION 1* (*TOC1*) [9]. In Arabidopsis, the aforementioned genes are involved in floral pathway integration (*LFY* and *SOC1*) [10], the determination of meristem identity (*LFY* and *AP1*) [11,12], inflorescence meristem identity (*TFL1*) [13], the sucrose flowering pathway (*ADG1*) [14], the gibberellic acid pathway (*GAI*) [15], and regulating circadian rhythm (*TOC1*) [16]. These homologues provide a simple framework onto which additional layers of complexity can be added as more flax flowering-time genes are identified. Using the highly curated flax reference genome [17,18], we can identify additional flowering homologues and examine the expression of putative flowering genes in specific tissues associated with the transition to flowering. This approach to deciphering the complex regulatory network of flowering time has been effective in numerous species, including crested wheatgrass [19], Moso bamboo [20], and litchi [21].

Our overall objective was to establish a template for the genetic regulatory control of the flowering transition in flax. We first identified flax homologues of 236 known Arabidopsis flowering genes and found that most (87%) occur in multiple copies, which is likely a result of ancient duplication events in flax [17]. The shoot apical meristem (SAM) is where all the shoot tissue is derived, and at the onset of the reproductive transition, the SAM changes to an inflorescence meristem from which the floral primordia are formed, and where many flowering genes are expressed. To target this tissue, we used an RNA-seq approach to investigate the transcriptome in the SAM of ‘Royal’ flax, a cultivar that we selected because of its historical use in flowering-time studies [6,7,8,22]. To collect expression data related to the early signals that determine the timing of the floral transition in flax, we hand-dissected shoot tips to collect SAM-enriched tissue, at three vegetative time points (10, 15, and 19 days after planting (dap)) and at 29 dap (when we first visibly observed production of reproductive tissue from the SAM). With this approach, we confirmed the expression of many putative flowering genes and differential expression of a subset of these with representatives from all the major flowering pathways. We determined that differentially expressed genes (DEGs) are enriched in transcription factor (TF) families that play a role in meristem identity and the floral transition, such as the MADS and SBP families. Finally, we compared the expression profiles of uncharacterized flax genes to those of DEG flowering clusters and identified candidates for novel flax flowering-time genes.

## 2. Results

### 2.1. Mapping of RNA-Seq Reads Generated Expression Estimates for 70% of Predicted Flax Genes

We used an RNA-seq approach to quantify transcript abundance in the SAM of ‘Royal’ flax at four time points (10, 15, 19, and 29 dap) and successfully generated many high-quality reads. Illumina paired-end sequencing produced an average of 26.2 million reads per sample (13.1 million read pairs) (Table 1). After trimming adapter sequences and low-quality nucleotides from the ends of the reads, we retained an average of 25.2 million reads per sample (12.6 million read pairs) with an average mapped length of 235.1 base pairs per read pair (Table 1). Approximately 92.1% of the trimmed read pairs were uniquely aligned to genes in the CDC Bethune reference genome and a small percentage (4.1%) were aligned to multiple loci (Table 1). We generated an MDS plot to assess the relationship between samples from different time points, and replicates of samples from the same time point (Figure S1). We observed overlapping of replicates from the same time point, indicating the absence of a batch, or replicate, effect; moreover, we noted a clear separation between samples from 10 dap and 29 dap. Samples from 15 and 19 dap clustered closer to each other than samples from any other two time points.

**Figure 1:**
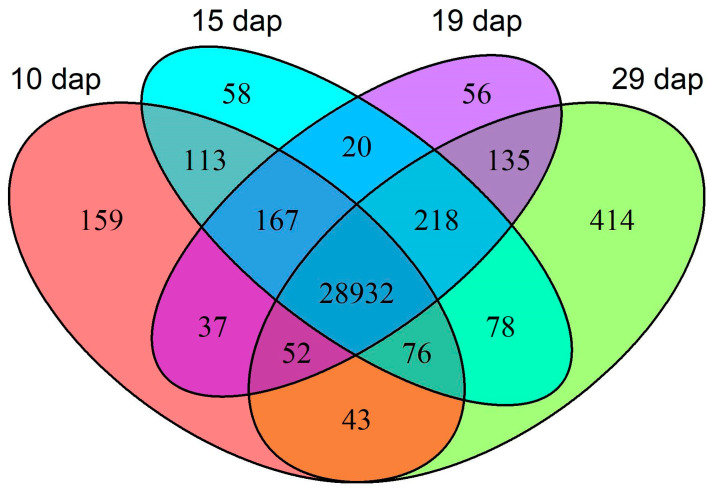
Number of expressed genes at 10, 15, 19 and 29 dap. Of a total 30,558 expressed genes, 28,932 were expressed in all treatments. Fewer genes were expressed in one, or a subset, of treatments.

We determined each gene’s time-point-specific expression using reads per kilobase of transcript per million mapped reads (RPKM) where concordant read pairs were used as ‘reads’ or ‘fragments’. We considered genes with RPKM ≥ 0.3 as expressed [23,24], and thus, detected the expression of 72% of the genes (30558 of the 42277 genes to which we made alignments) (Table S1). We assessed the relative levels of each gene’s expression and found the largest proportion of genes (~37%) had medium levels of expression (1 ≤ RPKM < 10) (Figure S2), ~23.5% and ~2.5% were expressed at high (10 ≤ RPKM < 100) or very high (RPKM ≥ 100) levels, respectively, and ~7% were expressed at low levels (0.3 ≤ RPKM < 1). For 95% of the expressed genes, we detected expression at all time points, with only a small percentage (~2%) being expressed at just a single time point (Figure 1; Table S1). Generally, we observed the highest RPKM in genes expressed at all time points (average RPKM of 22.43), as compared to the generally low expression of genes expressed at only a single time point (average RPKM of 0.63) (Figure S3). A small number of genes were expressed specifically over two consecutive time points; we counted 113, 20, and 135 genes expressed at 10 and 15 dap; 15 and 19 dap; and 19 and 29 dap, respectively. We detected expression of a slightly higher number of genes specifically expressed at three consecutive time points, which appeared to be either downregulated prior to 29 dap (i.e., the 167 genes expressed specifically at 10, 15 and 19 dap), or upregulated after 10 dap (i.e., the 218 genes expressed specifically at 15, 19 and 29 dap). A small number of genes (286) were expressed specifically at a combination of non-consecutive time points (10 and 19 dap; 10 and 29 dap; 15 and 29 dap; 10 and 15 and 29 dap; or 10, 19 and 29 dap). These non-consecutively-expressed genes exhibited low levels of expression, and these patterns may have resulted from variability in the sampling or technical variability, or they could just be random fluctuations in gene expression. 

### 2.2. Flax Genome Includes Homologues of 74% of Arabidopsis Flowering-Time Genes

Previously, researchers identified flax homologues of Arabidopsis genes using a BLASTP search [17]. From this published list, we counted 39,336 flax homologues of 14,771 Arabidopsis genes (~54% of Arabidopsis protein-coding genes) (Table S2). From this complete set of homologues, we focused the remainder of our analysis on homologues of Arabidopsis flowering genes. Bouché [4] and Fornara [5] described 318 genes regulating flowering time in Arabidopsis, and we determined that 236 (74%) of them have flax homologues (Table S2). We found multiple copies of genes previously identified in flax [6,8], which include *LFY* (Lus10016732.g and Lus10022427.g) and *SOC1* (Lus10036542.g, Lus10036543.g, and Lus10041385.g). We also noted homologues of many important flowering genes that have been previously unreported in flax to our knowledge, such as *FRUITFULL* (*FUL*) (Lus10007983.g, Lus10021140.g, and Lus10034662.g), *PENNYWISE* (*PNY*) (Lus10004688.g, Lus10021498.g, and Lus10022599.g) and *POUND-FOOLISH* (*PNF*) (Lus10016110.g, Lus10021452.g, and Lus10040256.g). The complete list includes genes from all major inductive pathways, floral pathway integrators, floral meristem identity genes, and genes involved in floral organogenesis.

We found that most homologues of the 236 flowering genes are present in multiple copies (at least two) in flax; ~45% are present in two copies; ~42% are present in more than two copies (3–16 copies, and one gene with 40 copies); and only ~13% occur as a single copy. This rate of duplication is higher than in non-flowering genes, where we found that ~20% occur as a single copy and ~80% have at least two copies (χ2 (1, N = 14,771) = 7.07, *p* = 0.007). In total, considering multiple copies, we identified 722 flax flowering-gene homologues (Table S2). Of these, ~80% were expressed during at least one time point, with 75% expressed at all time points and only ~3% expressed at one specific time point (Table S1). We identified a small number of genes with particularly high copy numbers (Table S2). Four of these were homologues of genes belonging to the gibberellic acid flowering pathway [25,26,27]: (1) 16 copies of *GA REQUIRING 2* (*GA2*); (2) 16 copies of *RGA-LIKE PROTEIN 1* (*RGL1*); (3) 12 copies of *RGA-LIKE PROTEIN 2* (*RGL2*); and (4) 11 copies of *REPRESSOR OF GA1-3 1* (*RGA1*). We also identified 40 homologues of *REDUCED VERNALIZATION RESPONSE 1* (*VRN1*), a gene with multiple roles in regulating flowering time in Arabidopsis and that involves both vernalization-requiring and vernalization-independent pathways [28]. These high copy number genes are not unique to flax flowering-gene homologues, as we also observed several non-flowering gene homologues with exceptionally high copy numbers (i.e., 76 flax homologues of AT5G36930, which encodes a disease-resistance protein).

We were unable to identify homologues for 82 Arabidopsis flowering-time genes (Table S3). This group of genes includes representatives from all major flowering pathways and some of the key players in Arabidopsis flowering. *CONSTANS* (*CO*) and *TEMPRANILLO 1* (*TEM1*), for instance, are essential for proper photoperiodic flowering responses in Arabidopsis, yet they lack a homologue in flax [29]. However, we did find homologues of genes related to these that are involved in flowering in Arabidopsis, including two *CONSTANS-LIKE 5* (*COL5*) homologues (Lus10015619.g and Lus10037636.g), and one *TEMPRANILLO 2* (*TEM2*) homologue (Lus10034276.g) [29,30].

### 2.3. Approximately 30% of Flax Genes Are Differentially Expressed in the SAM

**Figure 2:**
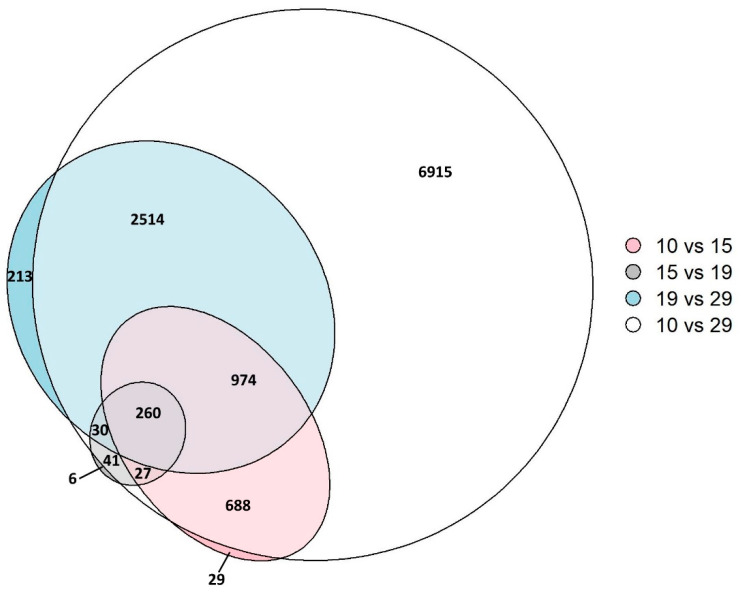
Number of DEGs between time points. A Venn diagram depicting the number of genes that are differentially expressed between neighbouring time points and/or between 10 and 29 dap, as well as the intersection between them. For example, there are 6915 genes differentially expressed only between 10 vs. 29 dap, and there are 2514 genes differentially expressed between both 10 vs. 29 dap and 19 vs. 29 dap. Due to the nature of area-proportional Venn diagrams, not all DEGs are shown, as the image is a visual approximation. Thus, although it is not depicted, there is also a single gene differentially expressed between both 10 vs. 15 dap, and 15 vs. 19 dap.

We observed differential expression of 12,130 genes (39.7% of the 30,558 expressed genes) (Table S4). Using a Venn diagram, we visually assessed the number of genes differentially expressed between neighbouring time points (i.e., 10 vs. 15 dap, 15 vs. 19 dap, and 19 vs. 29 dap), and the overlap between them (i.e., the number of genes differentially expressed between multiple pairs of time points) (Figure 2). We also included genes that were differentially expressed between 10 and 29 dap to consider genes that significantly changed in expression over the course of the study, but had only non-significant changes between neighbouring time points. Many genes were differentially expressed between 10 vs. 15 dap (1979) and 19 vs. 29 dap (3991). Not surprisingly, only a relatively small number of genes (365) exhibited differential expression between 15 vs. 19 dap (the most visibly similar time points), while the largest proportion of DEGs (94%) were differentially expressed between 10 vs. 29 dap (the most visibly different time points). A small number of genes changed expression only between neighbouring time points (29, 6, and 213 DEGs between 10 vs. 15; 15 vs. 19; and 19 vs. 29 dap, respectively). In contrast, 57% of DEGS (6915 genes) were differentially expressed specifically between 10 and 29 dap (Table S4). We also detected differential expression of 4535 genes between multiple pairs of time points. For example, 2514 genes had expression changes between both 19 vs. 29 dap, and 10 vs. 29 dap. Finally, 260 genes were differentially expressed between all three sets of neighbouring time points (i.e., 10 vs. 15; 15 vs. 19; and 19 vs. 29 dap), as well as 10 vs. 29 dap.

### 2.4. Gene Ontology (GO) Terms Related to Reproduction Are Enriched in Sets of Upregulated Genes

Separately for each pair of the time points, we tested for enrichment of individual GO terms within: (1) genes that are upregulated and (2) genes that are downregulated. We identified enrichment of many of the same GO terms related to reproduction in the sets of upregulated genes (Table S5). For example, in all sets of upregulated genes, except those upregulated between 15 and 19 dap, where we identified very few DEGs, we observed enrichment of terms for “Flower Development” (GO:0009908), “Floral Whorl Development” (GO:0048438), and “Floral Organ Development” (GO:0048437). We also found enrichment of GO terms related to the development of specific floral organs, such as “Anther development” (GO:0048653), “Androecium development” (GO:0048466), and “Stamen development” (GO:0048443) in genes with significantly increased expression between 10 vs. 19 dap, 10 vs. 29 dap, 15 vs. 29 dap, and 19 vs. 29 dap (Table S5). We observed that downregulated genes were significantly enriched for terms related to photosynthesis (Table S5). For example, “Photosynthesis” (GO:0015979), “Plastid Organization” (GO:0009657), and “Chlorophyll Metabolic Process” (GO:0015994), among others, were enriched in all six sets of downregulated DEGs.

### 2.5. DEGs Include Homologues from All Major Flowering Pathways

**Figure 3:**
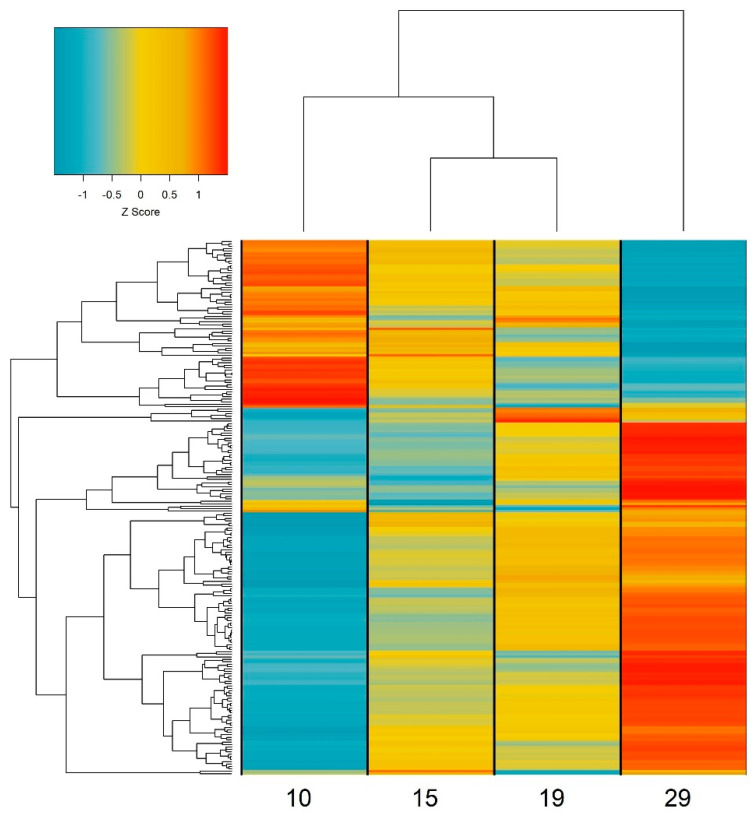
Heatmap and hierarchical clustering of flowering DEGs at 10, 15, 19, and 29 dap. Clustering along the left−hand side indicates relatedness of genes based on their expression pattern, and clustering along the top indicates relatedness of time points. Colour scale is based on Z−scores of TMM−normalized read counts, with blue indicating lower levels of expression and red indicating higher levels of expression.

Within the 12,130 flax DEGs, we identified 220 putative flowering genes (Table S4). These genes predominantly fall into one of two groups: increasing or decreasing expression patterns with plant developmental age (Figure 3). Specifically, we found that 148 putative flowering DEGs exhibit only significant increases in expression, while 69 only significantly decrease in expression (Table S4). Just three genes displayed variable changes in expression between time points (i.e., they showed significant increases between some time points and significant decreases between others), which may indicate a functional role in establishing both early flowering signals as well as later signals for flower development, or may result from genes having an additional role beyond the regulation of flowering time.

As with the entire set of DEGs, we found that most flowering DEGs (204/220) significantly changed in expression between 10 and 29 dap; 139 (68%) of these had higher expression at 29 dap than at 10 dap, and 62 (30%) were differentially expressed only between 10 and 29 dap (Table S4). We found that fewer putative flowering genes exhibited differences between the other time points (Table S4), with 36, 10, and 74 putative flowering-time genes varying in expression between 10 vs. 15 dap, 15 vs. 19 dap, and 19 vs. 29 dap, respectively. 

We identified members from all major flowering pathways amongst the putative flowering DEGs (Table S4). This list includes homologues of: *LATE ELONGATED HYPOCOTYL* (*LHY*) (Lus10012602.g, Lus10010100.g, Lus10005134.g, and Lus10030183.g) (photoperiod and circadian rhythm pathways); *SQUAMOSA PROMOTER BINDING PROTEIN-LIKE 9* (*SPL9*) (Lus10012020.g, Lus10021034.g, Lus10016275.g, and Lus10023818.g) (autonomous and aging pathways); *GA INSENSITIVE DWARF 1B* (*GID1B*) (Lus10027969.g and Lus10008189.g) (gibberellic acid pathway); *VRN1* (Lus10009688.g, Lus10016777.g, Lus10012041.g, Lus10023843.g, Lus10014359.g, Lus10027940.g, Lus10038189.g, Lus10026067.g, Lus10014528.g, Lus10017970.g, Lus10019652.g, and Lus10008529.g) (vernalization pathway); and *SUCROSE SYNTHASE 4* (*SUS4*) (Lus10020506.g) (sugar pathway). It also includes floral pathway integrators, such as *SOC1* (Lus10036542.g, Lus10041385.g, and Lus10036543.g), and floral meristem identity genes, such as *AP1* (Lus10026679.g, Lus10005081.g, Lus10004637.g, Lus10034370.g, and Lus10017871.g). We also identified differential expression of a small number of genes involved in determining floral organ identity, such as *SEP3* (Lus10015765.g) (Table S4).

### 2.6. DEGs Are Enriched in Transcription Factor (TF) Families Involved in Flowering

**Figure 4:**
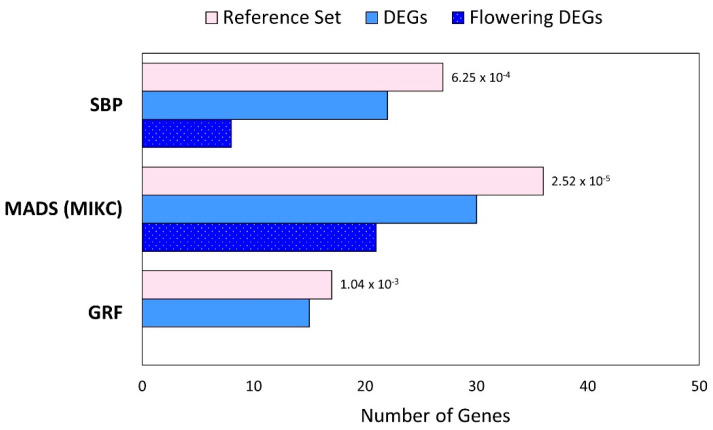
Enrichment of DEGs in TF families. Three families with a significant enrichment of DEGs are shown. The reference set (pink bars) indicates the number of expressed flax homologues from each family, and blue bars indicate the numbers of DEGs. The number of flowering DEGs are also shown (blue dotted bars). *p*-values indicate the significant overrepresentation of DEGs within each TF family.

Using a hypergeometric test, we determined that three TF gene families with an established role in regulating flowering have a significant enrichment of DEGs (Figure 4). We determined this by first identifying putative flax TFs, which was based on homology to Arabidopsis TFs [17,31]. We identified 2680 homologues of Arabidopsis TFs, together belonging to 57 families (Table S6). Of the putative TF-encoding genes, we detected differential expression of 789 that belong to 55 TF families (Table S6). We found significant enrichment of DEGs within three TF families (Figure 4): (1) GRF (Growth Regulating Factor); (2) MADS (*MCM1/AGAMOUS/DEFICIENS/SRF*) from MIKC class (MADS intervening keratin-like and C-terminal class; and (3) SBP (*SQUAMOSA promoter binding protein-like*) families. 

A total of five differentially expressed GRF TF-genes were detected, none of which encode putative flowering genes (Table S6). We identified 30 putative MADS (MIKC) TF-encoding genes that were differentially expressed (40% of all flax predicted MADS-MIKC genes). Of these 30 MADS DEGs, 21 (70%) encode flowering gene homologues. The majority of these (15/21 genes) increased in expression between 10 and 29 dap, including homologues of *SEP3* (Lus10015765.g); *SOC1* (Lus10041385.g, Lus10036543.g, and Lus10036542.g); *AP1* (Lus1004637.g, Lus10005081.g, Lus10017871.g, Lus10034370.g and Lus10026679.g); and *FUL* (Lus10007983.g, Lus10021140.g, and lus10034662.g). The remaining six genes gradually decreased in expression over time, which suggests that they may act as repressors of flowering and include a homologue of the floral repressor *FLOWERING LOCUS C* (*FLC*) (Lus10015766.g). 

We also identified 29 putative SBP TF-encoding genes and found 22 were differentially expressed. We also found that the SBP TF family contains differentially expressed flowering gene homologues (Figure 4; Table S6). Of the 22 SBP DEGs, eight (36%) were putative flowering genes, which include single copies of *SQUAMOSA PROMOTER BINDING PROTEIN-LIKE 4* (*SPL4*) (Lus10039846.g) and *SQUAMOSA PROMOTER BINDING-LIKE 5* (*SPL5*) (Lus10018610.g), and multiple copies of *SQUAMOSA PROMOTER BINDING PROTEIN-LIKE 3* (*SPL3*) (Lus10013999.g and Lus10015421.g) and *SPL9* (Lus10012020.g, Lus10016275.g, Lus10023818.g and Lus10021034.g). All 22 SBP TF homologues increased in expression between 10 and 29 dap.

### 2.7. Uncharacterized Flax Genes Include Candidates for Novel Flowering-Time Genes

We identified 468 candidates for novel flax flowering-time genes using a two-step process. Using a K-means approach, we first determined an optimal cluster number of two and clustered the flowering DEGs accordingly. These two clusters had opposing expression trends, with cluster one increasing in expression from 10 to 29 dap (151 genes), and cluster two decreasing (69 genes) (Figure S4). 

Second, we selected the 2516 uncharacterized flax DEGs that either lacked an Arabidopsis homologue (1075 genes) or that had an Arabidopsis homologue with an unknown function (1441 genes), and identified correlations between their individual expression patterns and the corresponding average expression pattern of either K-means flowering gene cluster. Using a threshold of R > 0.99, we identified 468 uncharacterized flax DEGs that shared an expression pattern with one of the flowering DEG clusters; 246 DEGs shared a pattern of increased expression with the genes in cluster-one, and 222 genes shared a pattern of decreasing expression with genes in cluster-two (Table S7).

### 2.8. RT-qPCR Validation of Gene Expression

**Figure 5:**
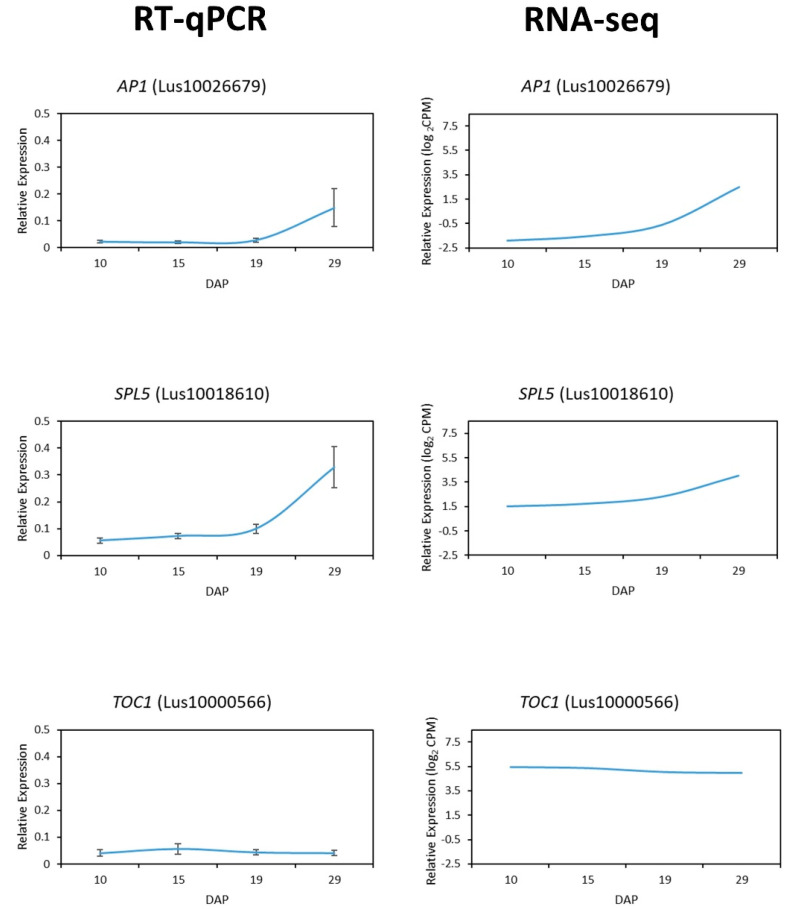
Expression of *AP1*, *SPL5*, and *TOC1* using normalized RNA−seq and RT−qPCR data. Validation of RNA−seq expression was conducted by comparing patterns of expression derived from RT−qPCR to those obtained from our RNA−seq analysis for three important flowering−time genes: *AP1*, *SPL5*, and *TOC1*. Data plotted for RT−qPCR are expressed as 2^−ΔCt^ and data plotted for RNA−seq are expressed as log_2_CPM. Normalization for RT−qPCR was performed using the reference gene *glyceraldehyde 3−phosphate dehydrogenase* (*GAPDH*).

To validate the RNA-seq results for DEGs, we selected three genes that are important regulators of flowering time—*AP1* (Lus10026679), *SPL5* (Lus10018610), and *TOC1* (Lus10015720)—and performed RT-qPCR (Figure 5). We used the normalized data from both RNA-seq and RT-qPCR to examine the expression between time points and found that the two approaches yield consistent patterns.

## 3. Discussion

A tremendous amount of research aimed at dissecting the Arabidopsis flowering regulatory network has resulted in the identification of more than 300 flowering-time genes [4,5], and researchers have found that many of them are conserved in various species [32,33,34,35,36,37]. Due to the breadth of information available on the Arabidopsis flowering gene network and the evolutionary conservation of many flowering genes across species, studies frequently rely on the Arabidopsis flowering regulatory network as a template to identify flowering genes in other species. For example, establishing the genetic flowering network of several legumes (*Medicago truncatula*, *Glycine max* and *Lotus japonica*) was achieved by identifying homologues of known Arabidopsis flowering genes [35]. Similarly, in wheat (*Triticum aestivum*) and barley (*Hordeum vulgare*), initial efforts to identify candidate flowering genes were accomplished via comparison to Arabidopsis [36]. This approach is beneficial as it allows an entry point through which we can begin to build a species-specific description of the network. Here, we present a global view of the pathways and genes that control the transition to flowering in flax, where we have identified 722 homologues of Arabidopsis flowering-time genes—220 of which exhibited differential expression during the reproductive transition—in addition to 468 novel flowering-time gene candidates. The results of our study have improved the resolution of the flax genetic flowering network, which will assist in the breeding of earlier-flowering varieties to expand the northern growing range of flax in Canada.

### 3.1. Genome-Wide Scan Reveals Flax Homologues of Most Arabidopsis Flowering-Time Genes

To identify putative flax flowering-time genes, we employed several approaches. First, we scanned the set of predicted flax genes from the reference sequence archived at Phytozome to identify homologues of known Arabidopsis flowering-time genes. We took this initial approach to identify candidate flowering genes in flax without imposing the specifications that accompany tissue- and time-point-specific gene expression, and to explore the extent of duplication amongst predicted flowering genes. Our study is the first, to our knowledge, to identify putative flowering genes in flax on a genome-wide scale. We present a comprehensive list of 722 flax homologues, corresponding to 236 of the known Arabidopsis flowering-time genes. These genes span all major flowering pathways—including the photoperiod, autonomous, gibberellic acid, vernalization, and age pathways—which include several floral pathway integrator genes, floral meristem identity genes, and a small number that also function in floral organ identity. We expected this relatively large degree of overlap in the flowering regulatory networks between flax and Arabidopsis as both species are hermaphroditic, predominantly inbreeding, have an annual growth habit, and have facultative long-day photoperiod responses [6,7,38].

Approximately 27% of Arabidopsis flowering-time genes lack substantial homology to flax genes. The absence of these homologues in the flax genome may indicate gene loss that is specific to flax or may be indicative of more ancestral lineage-specific gene loss. Alternatively, it could indicate that the genes in question evolved in Arabidopsis after the lineages that led to flax and Arabidopsis diverged. Some of the ‘missing’ genes may be functionally redundant to other flowering genes, which would have provided an opportunity for sequence divergence that led to their apparent loss in flax.

We found that some important Arabidopsis flowering genes, such as *CO*, are absent in flax. In Arabidopsis, the major role of CO is upregulating *FT* in leaves during inductive long days [39]. *CO* belongs to the *COL* gene family, which includes 17 members, each with two conserved domains (zinc finger region and a CCT domain) and divided into groups by sequence similarity [40,41]. Genes that function similarly to *CO* have been identified in other species [42,43,44,45]. For example, researchers found that the *Brassica napus* gene *BnCOa1* can complement a *co* mutant in Arabidopsis, revealing that the two genes are functionally equivalent [45]. Though we did not identify a direct homologue of *CO*, we did find genes with similarities to Arabidopsis *COL5*, which is classified in the same group of *COL* genes as *CO* and also functions in flowering [30,41]. Despite the stronger sequence similarity of these putative flax genes to *COL5* than to *CO*, without further investigation of the expression of these genes and functions of their proteins, we cannot rule out a *CO*-like role. The presence of *CO*-like genes may indicate functional complementation that resulted from loss of the original *CO* gene in flax. However, there are also other species, such as *Medicago truncatula*, that truly lack a *CO* homologue [46]. Sawa and Kay [47] suggested that instead of *FT* being regulated by CO, *FT* may instead be regulated directly via GI in Medicago. We previously identified homologues of *GI* and *FT* in flax and detected their expression in leaves [7]; these early expression analyses indicate the need for further investigation into the regulation of *FT* directly via GI in flax.

### 3.2. Putative Flax Flowering Genes Are Rich in Duplicates

Most plant species have undergone whole genome duplication events through their evolution [48], and flax is no exception. Flax is derived from an ancient polyploid, having undergone a paleopolyploidy event 20–40 million years ago [49] and a whole genome duplication ~5–9 million years ago [17]. Gene duplication is important for the development of novel genes, and following a duplication event, genes can have several fates: (1) one of the gene copies becomes a pseudogene and lacks function; (2) the copies diverge in function (neofunctionalization); (3) both copies retain a portion of their original functionality and, together, maintain the function of the ancestral gene (sub-functionalization); or (4) both gene copies maintain their original function, rendering the organism less susceptible to the effects of mutation in either copy [50,51,52,53,54]. 

Taking multiple copies of the same gene into account, we identified 722 putative flax flowering genes; only 13% represent genes with a single copy and 87% represent genes with at least two copies. This degree of duplication within the set of flowering gene homologues is higher than previous reports for the entire flax genome, which identified 9920 pairs of duplicated genes in the whole genome [17]. It is also higher than the degree of duplication in non-flowering genes, where we found that 80% have multiple copies. This may indicate that sub-functionalization of flowering genes following genome duplication in flax has been an important adaptive strategy. Notably, we found a particularly large copy number for homologues of a few genes in the gibberellic acid and vernalization pathways that deviate from the average copy number of two. For instance, we observed a disproportionately high copy number for *VRN1* (2.5 times more copies than the next highest copy number gene), a gene that, in Arabidopsis, encodes an AP2/B3-like TF [55]. This may represent an instance of lineage-specific gene family expansion, an important mechanism of adaptation [56,57]; however, another possibility is that some of the genes predicted to be homologues of *VRN1* are actually homologues of other closely related AP2/B3-like genes. If we assume that all copies are truly homologues of *VRN1*, we can look to their expression for evidence of a role in the floral transition. Of the 40 putative *VRN1* homologues, 12 were expressed similarly across all time points, 12 were differentially expressed between at least two time points, and 16 were not expressed. The 12 differentially expressed *VRN1* homologues may play a role in the transition to flowering, though additional research is required to verify the role of any flowering gene homologue in the timing of flowering. The remaining copies are likely to have diverged from their original function, be expressed solely outside of the SAM, or are silenced. *VRN1* provides just a single example of a duplicated gene with copies that seem to have diverged from their original function, but an in depth look at all putative flowering genes with multiple copies will likely reveal similar findings for additional genes.

### 3.3. Many Flowering Homologues Are Expressed, but Only a Small Number Are Differentially Expressed

After identifying homologues of Arabidopsis flowering genes, we wanted to determine which were expressed, and further, which were differentially expressed. Flowering-time genes are generally expressed in either, or both, the leaves and the SAM; however, previous studies indicate that the diversity and abundance of transcripts tends to be higher in SAM tissue [58]. To collect expression information on as many genes as possible, we selected SAM-enriched shoot-apex tissue for our analyses. Using high-throughput Illumina RNA sequencing, we quantified the expression of genes at four time points, the earliest being 10 dap (early vegetative development) and the most advanced being 29 dap (when we observed the first visible reproductive changes at the SAM). We detected the expression of ~72% of predicted flax genes. This is similar to gene expression during early development in Arabidopsis, where ~76% of genes are expressed in the SAM prior to the development of visible floral tissues [59], and soybean, where 73.5% of transcripts are expressed in the SAM just prior to development of the inflorescence meristem [58]. We noted that genes expressed at all time points had more variation in expression level, ranging from an average RPKM of 0.3 to 5870, while genes expressed at a single time point had a narrow and low range between 0.3 and 33. Genes expressed at all time points likely include housekeeping genes that are essential for proper cell function and the broader range of expression levels of these genes may indicate that they have more regulators than genes expressed at a single time point [60]. It is important to note that our data revealed a set of 18,474 genes that are expressed in the flax SAM, but not differentially expressed, and that this set likely includes housekeeping genes that are important for the maintenance of cellular functions. Some of these may be universally expressed in all cells, and a subset may be expressed specifically in the SAM. Comparison of expression of these genes in other tissues will help to resolve their biological role(s) and tissue specificity. Though we detected ~30,000 expressed genes, we filtered our dataset to only those genes encoding putative flowering-time gene homologues. Of the 722 flowering gene homologues, we detected expression of 80%, and for almost all putative flowering genes with multiple copies, at least one copy was expressed. 

We examined the expression of flowering gene homologues and found that ~30% were differentially expressed, and that they include representatives from all major flowering pathways. Some of these genes had expression changes between 10 and 15 dap, well before our first visual indication of the floral transition, which occurred at 29 dap. These differences in transcript abundance may represent some of the earliest molecular signals of the reproductive transition in flax. Some of these expression differences may describe physical changes occurring at the shoot tip, and we can look at the enriched GO terms to gain a better understanding of the biological processes that are affected by expression changes specifically at the SAM. For instance, in early upregulated genes (10 vs. 15 dap), over-represented terms described general processes of reproduction and the production of floral organs. In genes upregulated between 19 and 29 dap, however, there was an abundance of genes related to the development of specific reproductive organs, such as those assigned biological process terms related to the androecium, anthers and stamen. The change in GO terms describing the general process to the formation of specific reproductive organs likely reflects the physical changes occurring at the SAM, from the original timing of the transition from vegetative to inflorescence meristem, to the production of reproductive organs. It is safe to assume that some of the flowering gene homologues that lacked detectable expression, and changes in expression, are transcribed at time points and/or in tissues that were not captured in our study. Future expression studies that include more advanced time points and additional tissues will likely support a role in flowering for some of these homologues.

### 3.4. Many Putative Flowering Genes Display Expected Expression Patterns

In many instances, we observed expression patterns for flax flowering gene homologues that are consistent with their expected role as either a promoter or negative regulator of the floral transition. *SOC1*, for instance, integrates signals from a multitude of flowering pathways in Arabidopsis [61,62,63] and promotes the transition to flowering by increasing in expression steadily towards the determination of floral meristem identity [64]. Here, we identified three *SOC1* homologues (Lus10036543.g, Lus10036542.g, and Lus10041385.g) that all significantly increase in expression between 10 and 29 dap and, previously, a single flax homologue of *SOC1* was shown to increase throughout vegetative development towards the onset of floral meristem identity in ‘Royal’ flax [6]. *AP1*, which, in Arabidopsis, functions in both floral meristem identity and floral organogenesis [65,66], is upregulated after exogenous and endogenous signals are received and transmitted from pathway integrator genes [67,68,69]. We observed a similar pattern for five out of six flax *AP1* homologues that had significantly higher expression at 29 dap compared to 19 dap. The expression pattern of these genes, among many others, supports a role in the reproductive transition.

For putative flowering genes with multiple copies, we sometimes observed inconsistent expression patterns that suggest sub-functionalization or neofunctionalization of some copies. *TARGET OF EARLY ACTIVATION TAGGED 1* (*TOE1*), for example, which is a negative regulator of flowering time in Arabidopsis [70], has seven copies in flax; three of them decrease in expression with increasing plant developmental age, as would be expected if they function similarly to Arabidopsis *TOE1* [70]. The remaining four copies show small but significant increases in expression, which suggests divergence from their expected role. This type of divergence may indicate neofunctionalization, as has been observed previously in other species for some flowering genes [71,72,73]. For instance, divergence and neofunctionalization in homologues of *FT* have been observed in poplar [73], where *PtFT1* functions in reproductive development, and *PtFT2* functions in vegetative development.

### 3.5. Flowering-Related MADS and SBP TF Families Contain Many DEGs

We found enrichment of DEGs in the MADS (MIKC class), SBP, and GRF TF gene families, whose members participate in diverse developmental processes, the most relevant here being flowering [74,75,76]. The abundance of DEGs in these families, relative to others, suggests that they are particularly active during the time points used in our study and may play an important role in corresponding developmental processes, such as the onset of flowering, as in other species. 

The MADS and SBP TF families contain many known flowering genes [74,77] and the relationship between genes in these families is well-established in Arabidopsis. Researchers originally named SBP TFs for their interaction with *SQUAMOSA*—the *Antirrhinum majus* homologue of Arabidopsis MADS TF *AP1*—and they are generally known for acting upstream of several MADS genes [78,79]. For example, *spl3/4/5* triple mutants reduce the expression of MADS gene *AP1*, and *spl2/9/11/13/15* mutants reduce expression of MADS genes *AP1*, *FUL* and *SOC1* [80]. In the flax SAM, during early development, we determined that 40% of putative MADS-, and 72% of putative SBP-, encoding genes are differentially expressed, and that many of the DEGs encode putative flowering genes. The high degree of enrichment of DEGs within the MADS and SBP families simultaneously, particularly the presence of differentially expressed flowering genes within these families with expected patterns of expression, may result from conservation of the relationship between MADS and SBP TF families in flax and the conservation of their role in floral initiation in flax. 

Members of the GRF TF family function mainly in leaf and stem development, but a small number also participate in root and floral organ development [81,82,83,84,85]. The GRF family is relatively small, having 8–20 members in land plant genomes on average, and nine in *Arabidopsis thaliana* [76]. Our finding of 21 GRF homologues in flax is consistent with these previous findings. We observed differential expression of ~70% of the putative flax GRF genes but did not identify any flowering gene homologues amongst them. With the majority of GRF genes regulating the development of vegetative tissues, the observed enrichment of DEGS within the GRF family is likely a result of leaf and stem development, which occurs simultaneously with early transcriptional signals related to flowering. It is likely that the putative GRF-encoding DEGs include some of the main players in the development of stems and leaves between 10 and 29 dap in flax.

### 3.6. Flax Contains Potentially Novel Flowering Genes

The flax genome contains many homologues of Arabidopsis flowering genes but likely also novel flowering genes, as is the case in other species [86,87,88]. *OsMADS51*, for example, is a rice-specific promoter of flowering in short-day conditions that lacks an Arabidopsis homologue [86]. In chickpea, a comparison of transcriptome data with genome and UniGene data from other species led to the identification of 3362 chickpea-specific transcripts and 741 legume-specific transcripts, respectively [89], and a large proportion of lineage-specific genes are expressed specifically during flower development [87]. Additionally, in quinoa (*Chenopodium quinoa*), 459 genes have been identified as Chenopodium-specific, and researchers have defined 269 genes that are expressed only in the SAM as putative Chenopodium-specific flowering genes [90]. We found that approximately 1400 flax DEGs lack an Arabidopsis homologue; within this subset of genes, there may be some that are lineage- or species-specific and, thus, that are unique to flax or in the lineage that produced flax. We also identified 1075 flax genes whose Arabidopsis homologue is uncharacterized and whose function is currently unknown. Since the results of expression studies are dependent upon the time point(s) and tissue(s) used in the analysis, as well as parameters selected for high-throughput approaches to analysis (such as RNA-seq), there may be Arabidopsis flowering genes that remain uncharacterized, as indicated by Desphande et al. [91], who identified 76 novel Arabidopsis genes with expression highly correlated to that of *LFY* and *FLC*. The flax homologues of uncharacterized Arabidopsis genes may also include genes that do not function in flowering-time control in Arabidopsis but are novel flowering regulators in flax.

To identify candidate novel flax flowering genes, we compared the expression patterns of the uncharacterized flax genes to those of the flowering DEG clusters. A similar approach was used in *Medicago truncatula*, where researchers identified candidate genes involved in the production of saponins—which are defensive plant compounds—by looking at uncharacterized genes expressed similarly to those with a known role in saponin biosynthesis [92]. With this approach, we identified 468 uncharacterized genes that may regulate flowering in flax. Further analyses on these candidate flowering genes, and all putative flowering DEGs identified in our study, should be performed to investigate their biological role. Such studies should include determining whether the genes contain conserved domains of known flowering gene families; whether exogenous and endogenous flowering signals affect their expression; whether they interact with any known flowering genes; and whether knockout mutants have altered transitions to flowering and/or the development of any reproductive organs/structures. 

## 4. Materials and Methods

### 4.1. Plant Material and Growth Conditions

We grew plants from the flax cultivar ‘Royal’ in a Conviron growth cabinet in the Phytotron facility at the University of Saskatchewan (Saskatoon, SK, Canada). The materials and corresponding voucher specimens for ‘Royal’ flax (CN 113270) are available at Plant Gene Resources of Canada (Saskatoon, SK, Canada), under the conditions of the Multilateral System for Access and Benefit-sharing of the International Treaty on Plant Genetic Resources for Food and Agriculture. We planted five seeds per 4 L pot filled with a propagation mix of soil from Sungro^®^ Horticulture (Seba Beach, AB, Canada). We provided plants with Miracle-Gro 15-30-15 fertilizer supplemented with 0.1 g/L copper sulphate at the time of seeding and watered as needed, daily, throughout the duration of the experiment. The growth chamber was set to a day/night temperature cycle of 22 °C/17 °C and a day/night light cycle of 18 h/6 h. We applied two preventative biological treatments to protect flax plants against thrip damage. Biological controls were applied to pots as needed at the time of seeding (*Hypoaspis miles*) and during the growth of the plants (*Amblyseius cucumeris*).

### 4.2. Tissue Collection

We hand-dissected shoot apices to generate shoot apical meristem (SAM)-enriched tissue samples at 10 h after the start of the light period at 10, 15, 19, and 29 dap. We selected these time points as they represent distinct developmental morphologies and growth phases (Figure S5): (1) when the first true pair of leaves unfolded (10 dap); (2) when the sixth pair of leaves unfolded (15 dap); (3) at early stem elongation (19 dap); and (4) at the first visible detection of floral organ development at the SAM (29 dap). We examined individual plants for morphological markers immediately prior to tissue harvest to reduce developmental heterogeneity in each sample. At each time point, we randomly selected 50 of the remaining plants for tissue collection. We collected three replicate samples of 50 plants at each time point. We placed all tissue samples in liquid nitrogen immediately upon collection and stored them at −80 °C. We conducted the entire experiment three times, for nine replicates at each time point.

### 4.3. RNA Extraction and Illumina Sequencing

We ground frozen tissue samples in liquid nitrogen using a mortar and pestle. We extracted total RNA from approximately 100 mg of ground tissue using a Qiagen RNeasy^®^ Mini Kit and removed contaminating genomic DNA using DNase I treatment (Qiagen, Toronto, ON, Canada) (both as per the manufacturer’s instructions). We quantified RNA using the Qubit RNA BR assay kit (Thermo Fisher Scientific, Burlington, ON, Canada) (according to the manufacturer’s instructions) and assessed sample integrity and quality using an RNA 6000 Nano labchip on an Agilent 2100 Bioanalyzer (Agilent Technologies, Santa Clara, CA, USA). cDNA library construction, filtering for mRNA, and sequencing were performed at the National Research Council Canada (Saskatoon, SK, Canada). Briefly, cDNA library construction was performed using a TruSeq RNA sample preparation kit from Illumina (San Diego, CA, USA) and four µg of total RNA from each sample. Paired-end libraries were sequenced using the HiSeq 2500 Illumina platform. All raw sequences are available in the National Centre for Biotechnology Information Short Read Archive under BioProject ID PRJNA698991.

### 4.4. Quality Control, Read Alignment, and Tests of Differential Expression

We assessed the quality of raw, paired-end reads using *FastQC* (Version 0.11.5) [93]. We then trimmed reads using *Trimmomatic* (Version 0.36) [94] to remove Illumina adapter and ambiguous sequences; leading or trailing low quality bases with a quality Phred score less than 24; and reads with an average quality per base below 15 in a 4-base sliding window, or below a minimum length of 50 bases. Using *STAR* (version 2.5.3a) [95] we aligned clean, paired-end reads to genes in draft copies of the recently published CDC Bethune reference genome and annotation files [18], which were generously provided by Frank You prior to publication. We used previously published [17] flax gene names (provided as version 1.0) that were derived from a *BLASTP* between the flax and Arabidopsis proteomes, and that are published on Phyotzome [96,97]. We used default parameters within *STAR* to align reads to genes, apart from the following parameters: (1) --outFilterMismatchNmax set to ‘15’ and (2) –quantMode set to ‘GeneCounts’ so that the number of concordant read-pairs (“fragments”) were counted for each gene. We kept genes with greater than one count per million reads in one or more time points for further analyses, which were performed in *RStudio* (version 3.5.1) [98]. To normalize raw counts with the TMM method, we used the *calcNormFactors()* function in the Bioconductor package *EdgeR* [99]. Gene expression levels were expressed as counts per million (CPM) using the *EdgeR cpm()* function [99]. RPKM was calculated using the *EdgeR rpkm()* function [99] using the number of concordant read pairs (‘fragments’) and the TMM normalized libraries as inputs. Based on previous research [23,24], we considered genes as being expressed at a given time point if they had an average RPKM ≥ 0.3. Prior to testing for differential expression, we calculated dispersion estimates, which were based on shared information across genes (i.e., common dispersion) using the *estimateGLMCommonDisp() EdgeR* function [99]. We used the *EdgeR glmLRT()* function to determine differential expression of genes between all pairs of time points. These results were adjusted for multiple testing using a false discovery rate correction [100], and we considered genes differentially expressed when FDR was *p* < 0.05.

### 4.5. Validation of Expression Patterns via Quantitative-PCR

To validate our RNA-seq results, we used RT-qPCR to determine expression patterns for three genes: *AP1* (Lus10026679), *SPL5* (Lus10018610), and *TOC1* (Lus10015720). The ‘Royal’ flowering-time gene sequences used were from the recently published flax genome, as described in the section ‘Quality Control, Read Alignment, and Tests of Differential Expression’ above. Forward and reverse primers, as well as probes (IDT) were designed to complement unique regions of each of the *AP1*, *SPL5* and *TOC1* flax homologues. Probes for homologues of the same gene were labelled with FAM or HEX to differentiate them from one another. A two-step RT-qPCR protocol was used. First, strand synthesis was performed on 2 μg total RNA using the Lunascript reverse transcriptase kit (NEB). Taqman reactions were performed using 1.5 μL of cDNA, 1 × SsoAdvance Probes Master Mix (BioRad), 300 nM forward and reverse primer, and 100 nM probe in a 15.0 μL total volume. The reference gene, *glyceraldehyde 3-phosphate dehydrogenase* (*GAPDH*), was co-amplified in the same tube for *AP1* and *TOC1*, but separately *for SPL5* due to interference with the reference assay. Thermocycling conditions were as follows: an initial denaturation at 95 °C 2 min; 40 cycles of 95 °C, 10 s; 55–58 °C, 10 s; and 72 °C, 15 s, with a plate read. The qPCR reactions were performed in white 384-well plates using a CFX384 thermocycler (BioRad, Mississauga, ON, Canada). 

### 4.6. Gene Ontology Enrichment Tests

We performed gene ontology singular enrichment analyses using *AgriGO* version 2.0 [101]. We used GO terms for flax as provided on the Phytozome website [17,97]. For each pair of time points, we tested for significant enrichment of GO terms in two sets of genes: (1) upregulated and (2) downregulated. We selected the hypergeometric test option, the complete set of GO terms, and a minimum of 10 mapping entries (that is, we required GO terms to be associated with at least 10 genes in the input list for that specific term to be tested for enrichment). A Benjamini and Hochberg [101] FDR correction for multiple testing was applied, and terms with *p* < 0.05 were considered significantly enriched.

### 4.7. Enrichment of Transcription Factor-Encoding Genes

As described above, sequence similarity between flax and Arabidopsis proteins was previously determined [17] and we inferred homology between the corresponding genes. We then identified putative flax transcription factors as the homologues of Arabidopsis transcription factors that are listed on PlantTFDB v 5.0 (Plant Transcription Factor Database) [31,102]. Within TF gene families, we determined enrichment by comparing the number of DEGs to the number of expressed genes using a hypergeometric test. We applied a Benjamini and Hochberg [100] FDR multiple testing correction using the *RStudio* function *p.adjust()* [98] and we considered families with *p* < 0.05 significantly enriched with DEGs.

### 4.8. K-Means Clustering

We clustered DEGs with a K-means approach. We used Z-score-transformed values for genes at all four time points, individually (equation shown below), as input, and the *Kmeans()* function of the *amap* package [103] in *RStudio* version 3.5.1 [98]. We calculated Z-scores for each gene, at each time point, based on the following formula:

Z = (X − µ)/σ,(1)

where X = TMM-normalized log CPM, µ = mean TMM-normalized log CPM of all time points combined, and σ = Standard deviation of all time points combined. 

The optimal number of clusters was determined using the *NbClust* R Package using the following parameters: Euclidean distances; a minimum and maximum number of clusters set to two and ten, respectively; complete method; and ‘all’ indices [104].

### 4.9. Identification of Genes with Expression Patterns Correlated with Those of Flowering Genes

To identify candidate novel flowering-time genes, we first identified uncharacterized flax, which we considered to be: (1) genes with an unknown function (i.e., flax genes whose Arabidopsis homologue has an “unknown” function and gene name, and (2) genes that lack a homologue in Arabidopsis. For both sets of genes, we calculated Z-scores.

For uncharacterized genes, we calculated Z-scores for each time point (as described above for single genes). For the two clusters of flowering DEGs, we calculated Z-scores for individual genes (as described above) and then calculated the average time-point-specific Z-score for each cluster. We used the average Z-scores at each time point for further analyses. The similarity in the expression patterns over time points between the individual uncharacterized genes and the DEG flowering clusters was determined using Pearson Correlations, which we calculated using the *cor()* function in *RStudio* version 3.5.1 [98]. We considered genes whose expression patterns were correlated (r > 0.99) with the average time-point-specific expression from either cluster 1 or cluster 2 as candidates for novel flowering-time genes.

## 5. Conclusions

We characterized the changes in gene expression that occur in flax SAM tissue throughout the initial changes in meristem identity from vegetative to reproductive development, and identified many putative flax flowering genes, most of which are present in multiple copies. Using an RNA-seq approach and SAM-enriched tissue from four time points leading up to the development of visible reproductive tissue, we observed the expression of most putative flowering genes, and the differential expression of approximately one third. The DEGs include members of all major flowering pathways, floral pathway integrators, and floral meristem identity genes, and are enriched in TF gene families with well-established roles in the reproductive transition. Finally, we found uncharacterized flax genes that have similar expression patterns to the flax flowering gene homologues, and identified these as flax flowering gene candidates for further investigation. The results of this study provide a framework of the molecular flowering network in flax that will serve as a template for future investigations; these include those aimed at determining the functional role of flowering gene candidates and their regulation via environmental and endogenous signals. The results of this study expand our understanding of the genetic flax flowering network and will aid in the breeding of northern-adapted varieties.

## Figures and Tables

**Table 1:** Mapping of Illumina Paired-End Reads.

Sample ^1^	# Reads Sequenced	# Reads after Trimming	Average Mapped Length (Basepairs) ^2^	# Uniquely Mapped ^2^	% Uniquely Mapped ^2^	# Mapped to Multiple Loci ^2^	% Mapped to Multiple Loci ^2^
10 dap	24,475,121	23,533,548	236.0	10,492,564	92.2%	451,616	4.0%
15 dap	27,808,974	26,777,881	234.7	11,550,847	92.2%	502,468	4.0%
19 dap	26,290,312	25,230,207	235.7	11,638,642	92.2%	511,126	4.0%
29 dap	26,147,671	25,130,943	234.2	8,362,641	91.6%	520,766	4.2%
Average	26,180,519	25,168,145	235.1	10,511,174	92.1%	496,494	4.1%

^1^ Each sample includes averages from three experiments, with each experiment consisting of three replicates. ^2^ Value is based on pairs of concordant reads.

## Data Availability

Publicly available datasets were analyzed in this study. This data can be found here: NCBI Sequence Read Archive (SRA) repository under BioProject ID PRJNA698991.

## Supplementary Materials

The following supporting information can be downloaded at: https://www.mdpi.com/article/10.3390/plants11070860/s1, Figure S1: MDS plot using raw RNA-seq reads; Figure S2: Distribution of genes based on RPKM; Figure S3: RPKM in genes expressed at all time points vs. genes expressed at a single timepoint; Figure S4: Box and whisker plots of relative expression for flowering DEG clusters at 10, 15, 19, and 29 dap; Figure S5: Images of ‘Royal’ flax at 10, 15, 19, and 29 dap; Table S1: Expressed flax genes; Table S2: Flax flowering gene homologues; Table S3: Arabidopsis flowering genes without a flax homologue; Table S4: Differentially expressed genes; Table S5: GO enrichment results; Table S6: Putative flax transcription factors; Table S7: Candidates for novel and/or lineage-specific flowering genes.
