# Supplementary material for: Transcriptomic Analysis of Early Flowering Signals in ‘Royal’ Flax
Source: Plants (Basel). 2022 Mar 24;11(7):860. doi: 10.3390/plants11070860 (PMC9002848; doi:10.3390/plants11070860)
Supplement: Supplementary file 1 [file plants-11-00860-s001.zip › Supplemental Figures_Royal Manuscript_2022.pdf]

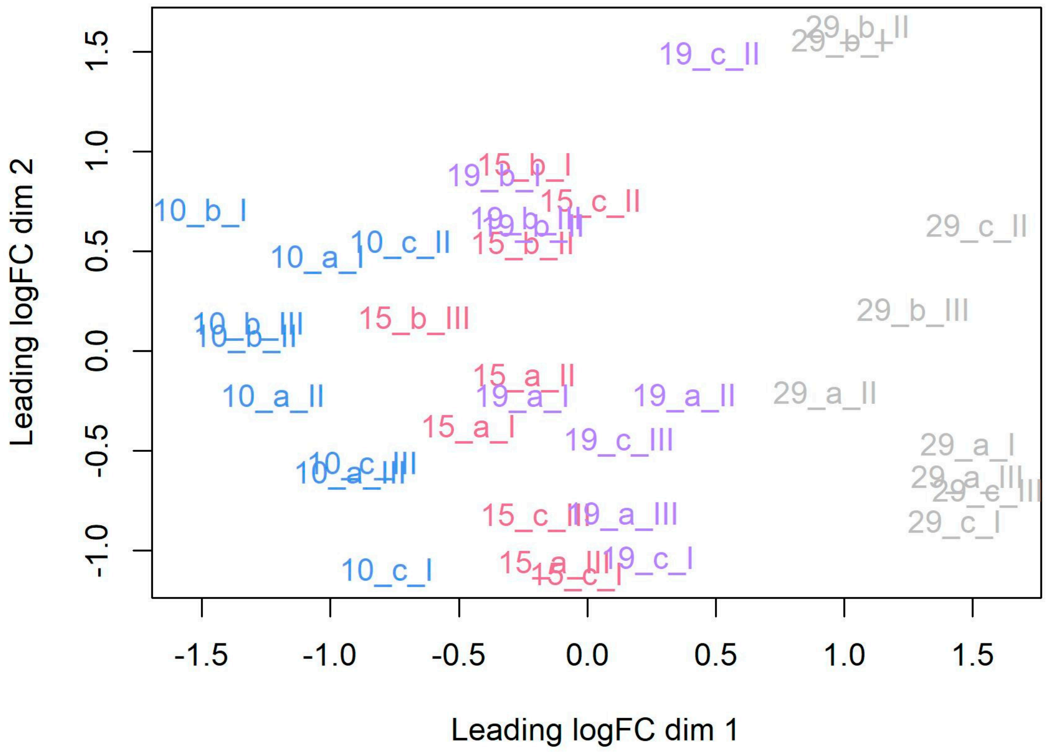

Supplemental Figure S1. MDS plot using raw RNA-seq reads. The distribution of points representing distances between samples based on $\log_2$ fold changes (logFC) indicates absence of a batch effect, similarity between reads from 15 and 19 dap, and clear separation of reads from 29 dap. Libraries representing four time points (10, 15, 19, and 29 dap), three experiments (a, b, and c), and three replicates from each experiment (I, II, and III) are plotted.

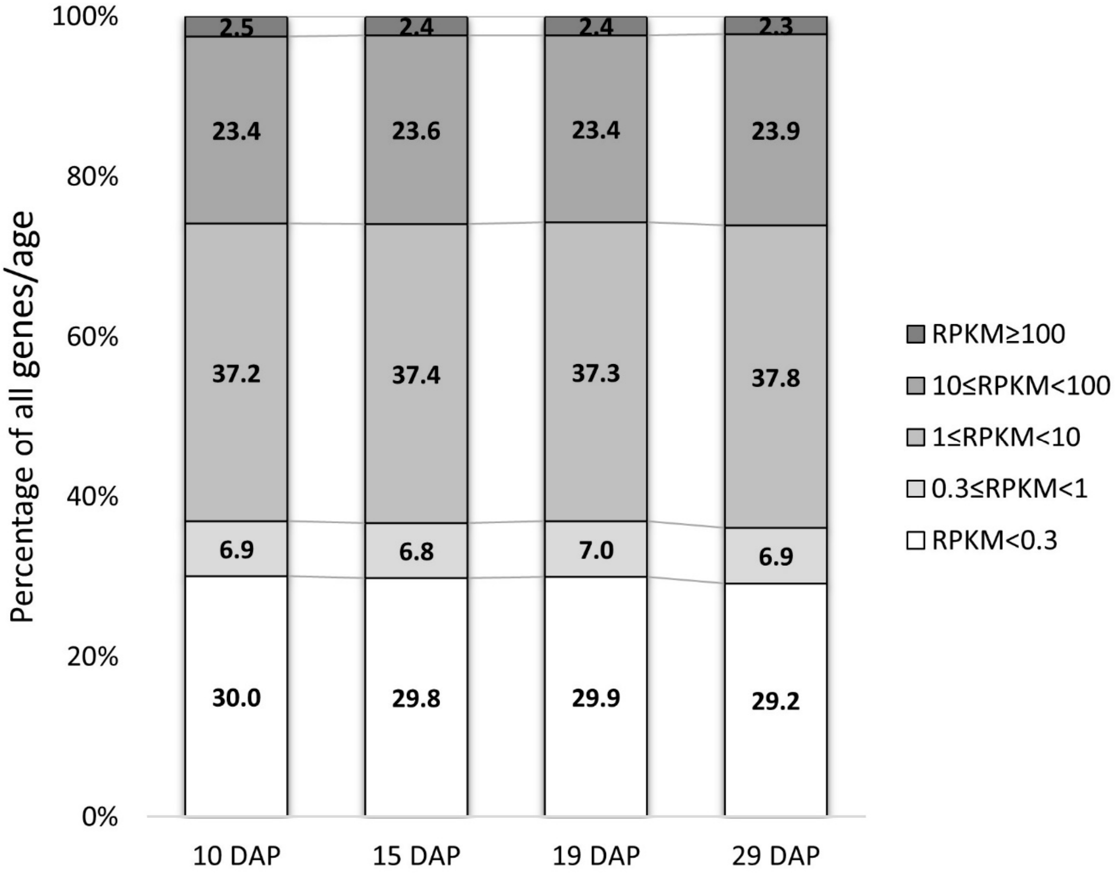

Supplemental Figure S2. **Distribution of genes based on RPKM**. Overall transcript abundance is similar across time points. Genes with RPKM < 0.3 were considered non-expressed within an time points and any genes with RPKM ≥ 0.3 were considered expressed. Of the expressed genes, we classified them as having low expression (RPKM <1), medium expression (1≤ RPKM<10), high expression (10 ≤ RPKM < 100), or very high expression (RPKM ≥ 100).

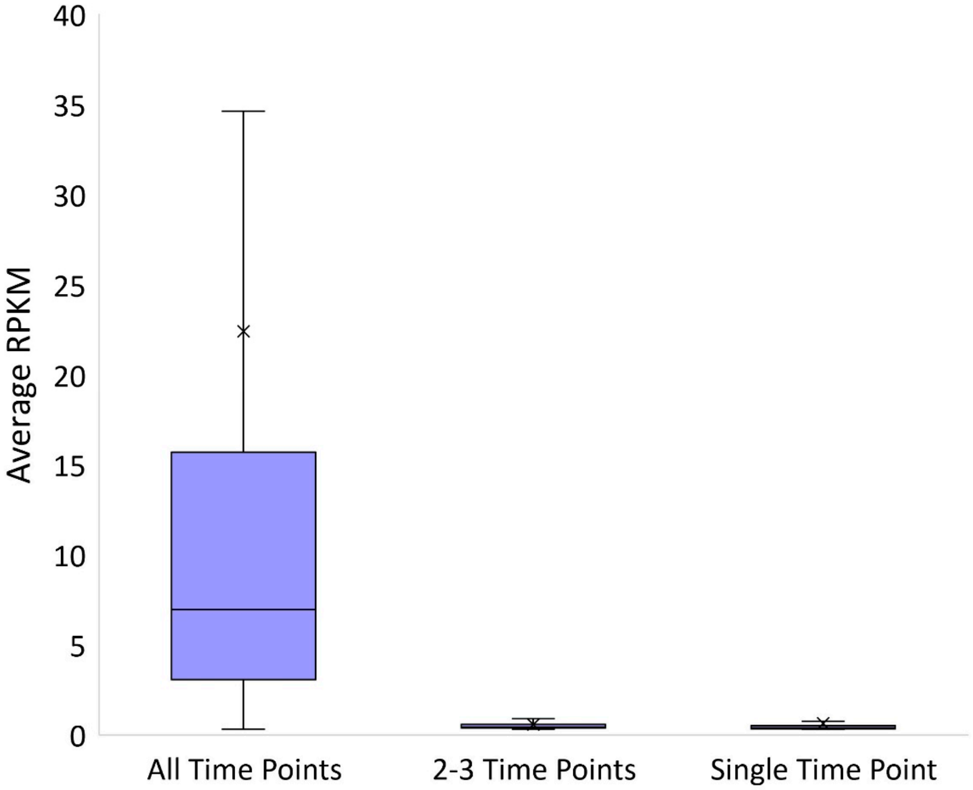

Supplemental Figure S3. RPKM in genes expressed at all time points vs genes expressed at a single timepoint. The range of RPKM values for genes expressed at all four time points greatly exceeds that of genes expressed at a single time point. Genes expressed at all time points are those with an average RPKM ≥ 0.3 at 10, 15, 19 and 29 dap. Genes expressed at 2-3 time points are those with an average RPKM ≥ 0.3 at any combination of two or three time points (for example, genes expressed at both 10 and 15 dap, or 10, 15, and 19 dap). Genes expressed at single timepoint were those with an average RPKM ≥ 0.3 at only one time point. Outliers are not indicated in the plot.

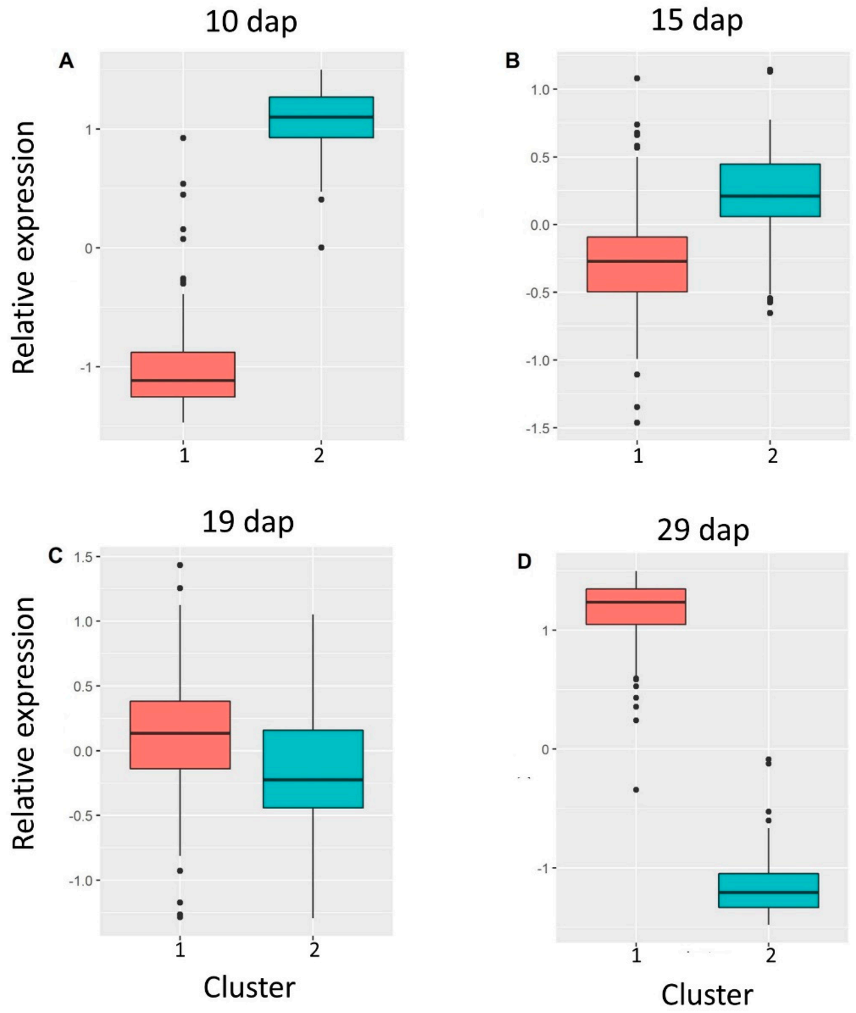

Supplemental Figure S4. Box and whisker plots of relative expression for flowering DEG clusters at 10, 15, 19, and 29 dap. These plots exhibit the differences in relative expression between the groups of genes belonging to clusters one and two at each of the four time points. A) 10 dap, B) 15 dap, C) 19 dap, and D) 29 dap. Expression of genes in cluster one is low at 10 dap, while expression of genes in cluster two is high. These patterns are reversed at 29 dap. Relative expression, as indicated along the y-axis, indicates Z-scores of normalized read counts.

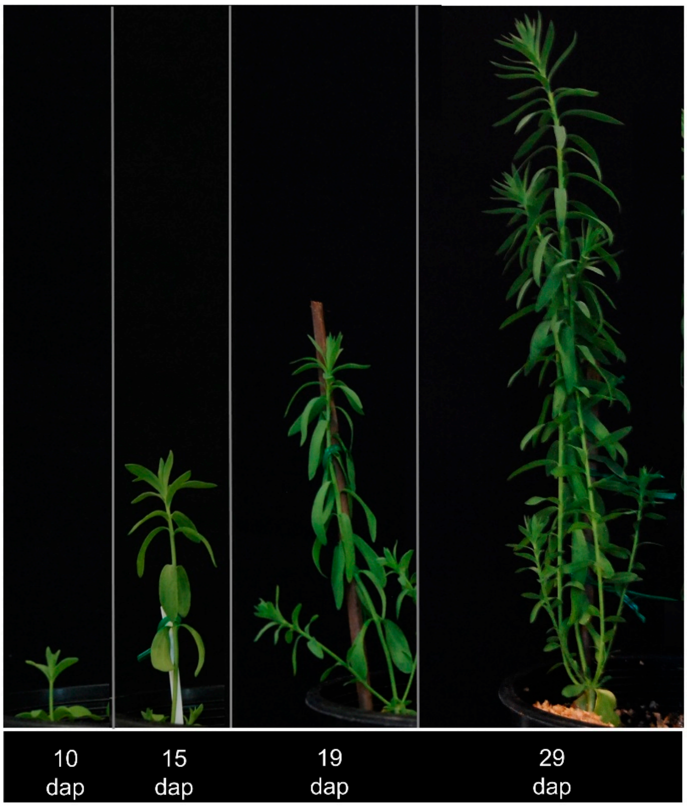

Supplemental Figure S5. Images of ‘Royal’ flax at 10, 15, 19, and 29 dap.
